# What Is an Organism?

**Published:** 1864-06

**Authors:** 


### Selections.

WHAT IS AN ORGANISM?—It used to be taught, and until recently was very generally believed, by the highest scientific authorities, as well as by unlearned persons, that certain phenomena occurring in living beings were in their very nature essentially distinct from any changes taking place, or which could be caused by man to take place, in inorganic matter. And, as a consequence, until very recently, *living* organisms have been regarded as in some way quite different from things inanimate. *Vital actions* were supposed to be different from *physical and chemical actions.*

But it has long been known that physical and chemical changes take place in living organisms, and of late it has been stated that these physical and chemical actions are the *only actions* which occur, and hence they are the "vital actions" which were believed in in a school gone by. If this be so, it would be correct to use the words "organism," "vital," &c., in cases in which it was not possible that ordinary life could be sustained, or it would be correct to give up the use of these words altogether. Inorganic matter in a certain state, but, as far as we know from experiment, utterly incompatible with life, has been said to exhibit *vital*

phenomena, although it can not *live* according to the ordinary sense of the word. On the other hand, things which undoubtedly do live, are said to exhibit changes resulting from the action of ordinary force. "Organizing force" is but another mode of heat.

If the development of heat, light and electricity results from vital action, and be characteristic of organisms, why should not a mass of inorganic matter which exhibits heat, light and electricity, be an organism? But it has not been shown that heat, light and electricity result from *vital action;* and, until this has been done, it must be more correct to regard them as *physical phenomena*, wherever they occur, than as vital phenomena when they are manifested in inorganic matter.

But, if it were true that there is no real distinction between *vital and physical and chemical actions*—if there were indeed, *no actions* occurring in living organisms peculiar to living organisms alone—let the use of all terms with which mysterious (vital) agencies are associated be abandoned; for, by retaining them, it is clear that we retard the real progress of science, and inculcate ideas which are not supported by facts, and which are really destitute of truth.

But it seems to me that many high authorities have rejected the idea of the existence of peculiar and unexplained actions (vital) in living beings, not upon insufficient evidence, but without any evidence to justify such a step; and it is not a little remarkable that not one of those who have accepted and taught these doctrines has ventured to describe what, according to his notion, takes place in living matter. It is right that these questions should be fairly investigated, and they are questions well worthy of attentive study, if the only object be to assign to many words now in common use, and employed very vaguely and in different senses, a definite and precise meaning.

Sir John Herschel has more than hinted that the term "organism" may be applied to matter even in a state of incandescence. Speaking of the willow-leaf like bodies discovered by Mr. Nasmyth upon the surface of the photosphere of the sun, he says:—These flakes, be they what they may, "are evidently the immediate sources of the solar light and heat by whatever mechanism or whatever processes they may be enabled to *develop*, and, as it were, *elaborate these elements from the bosom of the non-luminous fluid in which*

*they appear to float.* Looked at in this point of view, we can not refuse to regard them as *organisms of some peculiar and amazing kind;* and, though it would be too daring to speak of such *organization* as partaking of the nature of life, *yet we do know that vital action is competent to develop both heat, light and electricity.*"—(*Good Words*, 1863, p. 282.)

But the President of the British Association goes a step further, and even ventures to suggest the particular class of organisms with which these bodies may be compared. He thinks they are shaped like *diatoms!* "I have still to advert to Mr. Nasmyth's remarkable discovery that the bright surface of the sun is composed of an aggregation of apparently solid forms, shaped like willow-leaves or some *well-known* forms of Diatomaceæ, and interlacing with one another in every direction. The forms are so regular in size and shape as to have led to a suggestion from one of our profoundest philosophers of their being *organisms, possibly even partaking of the nature of life*, but, at all events, closely connected with the *heating* and *vivifying* influences of the sun." (Report of Sir William Armstrong's Address to the British Association, *Athenæum*, August 29th, 1863.) In the above quotations the italics are my own.

The word "organism" is here applied to sheets, flakes, or scales having some sort of solidity, about 1000 miles in length, and 200 or 300 in breadth, the temperature of which is far higher than would be sufficient to keep the most refractory metals in a state of complete fusion, if not to convert them into vapor. With true respect, but with the utmost earnestness, I venture to express the opinion that "organism" can not be properly applied to these bodies.

I will advance my objections *seriatim*, and I hope to be able to show that there are actions going on in every kind of living matter totally different in their nature to any actions which are known to occur in ordinary inorganic matter. I submit, in spite of many assertions, but *mere assertions* to the contrary, that we must still draw a most definite and well-marked distinction between mere physical and chemical changes, whether they occur in things that "live" or in inanimate inorganic matter; and *those peculiar "vital actions"* which alone occur in matter that is alive. In these papers it will be my object to attach a precise and definite meaning to the terms "vital," "life," &c., and I shall endeavor to state the arguments in favor of this view as simply and clearly as possible. The evidence which I shall bring forward, I think, justifies the inference that these solar bodies regarded as "organisms," differ from any known organisms in so many essential particulars as to render this term altogether inappropriate. I hold that this term "organism," can not be correctly applied to these solar bodies and also to living beings. From any known living beings these bodies differ in structure, in composition, in size and form, and in action; for it will be shown that, in every kind of living matter known to man, certain actions take place which can not possibly occur in the sun.

1. As to structure. Every living organism around us, and every elementary part of an organism, consists of matter in two very different states. It is not possible to find a piece of any living tissue as much as $\frac{1}{500}$th of an English inch in diameter, which exhibits uniformity of structure throughout.

In answer to this asserted difference in structure between living tissues and inorganic matter, it will be said—Corresponding differences in structure *are* observed; for example, the section of a plum-pudding stone, of granite, of certain homogeneous viscid substances subjected to the action of a voltaic current, &c., shows masses or spaces more or less isolated, exhibiting certain characters, in the substance of a matrix possessing characters and properties very different. Stones and brick embedded in mortar are like the "cells" embedded in the "intercellular substance" of which such a tissue as cartilage is composed. To this I answer as follows:

*a.* Active and constant changes take place in the small collections of matter of which the so-called "cells" of the living tissue are partly composed, while the stones embedded in the mortar undergo no change.

*b.* The intercellular substance of the living tissue is *not deposited* around the "cells" like mortar around the stones, but it is formed from them. All the matter forming the "intercellular substance," or "cell wall," was at an earlier period in the same state as that matter of which the so-called "cells" are constituted. Now the mortar can exist without the stones embedded in it, but no "intercellular substance" was ever formed without "cells." And mortar may be made first, and then stones may be embedded in it; but "intercellular substance" is never formed first, and "cells" forced into it, or caused to appear in it. Stones can never produce the mortar which surrounds them, but "cells" always exist before the "intercellular substance," and always take part in its production.

*c.* The soft granular matter of which the "cell" is composed during the living state gradually passes by continuity of structure into the surrounding material—termed "intercellular substance," in some tissues, "cell wall" in others.

*d.* The soft granular matter of which the "cells" are composed exhibits differences of appearance in different parts. And I shall adduce evidence to show that growth in these masses occurs from the centre. These zones have received arbitrary names, "Nucleolus," "Nucleus,"—but *names* are unimportant to the argument. No such differences, it need scarcely be said, exist in the stones. When the latter are embedded in the mortar no further change occurs; but it is easy to show that most active and important changes occur in the "cells" although they are surrounded by "intercellular substance."

*e.* The smallest particle of each little mass or cell of a living tissue *selects*, *forms*, *converts*, and can communicate these wonderful powers to inanimate matter which comes into contact with it, but the surrounding "intercellular subtance," or "cell wall," does not possess these wonderful powers which are peculiar and correctly termed VITAL powers. Hence no true analogy can be drawn between "cells" embedded in "intercellular substance" and bricks or stones embedded in mortar, until the bricks or stones make themselves out of matter differing from them and from the mortar in composition, multiply in number of their own accord, and form and deposit the mortar which surrounds them.

Before the bodies in the photosphere of the sun can be compared to organisms, it must be shown that they possess some sort of *structure*, or at least it should appear reasonable to assume that some such difference in structure as can be shown to exist in all living tissues without exception, was possible and probable. But all known organisms differ from these bodies and from every kind of inorganic matter in many particulars besides structure.—L. S. B., King's College, London, in *The Reader*.

